# Exploring the association between genetic polymorphisms in *FGFR2* and children’s facial morphology

**DOI:** 10.1186/s13005-026-00649-3

**Published:** 2026-08-15

**Authors:** Christian Rinnert, Svenja Beisel-Memmert, Peter Proff, Cristiano Miranda de Araujo, Maria Julia Pugsley Baratto, Camila Paiva Perin, Juliane Corá, Christian Kirschneck, Erika Calvano Küchler

**Affiliations:** 1 Department of Orthodontics, Medical Faculty, University Hospital Bonn, University of Bonn, Welschnonnenstr. 17, Bonn, 53111 Germany; 2 Department of Orthodontics, Medical Faculty, University of Regensburg, Regensburg, Germany; 3 School of Dentistry, University of Tuiuti of Paraná, Curitiba, Brazil

**Keywords:** FGFR2, Polymorphism, Photogrammetry, Facial Morphology

## Abstract

**Background:**

Studies on animal models and fibroblast growth factor receptor (FGFR2)-associated craniosynostosis syndromes suggest that FGFR2 plays a crucial role in craniofacial development. Therefore, the present exploratory study aims to investigate the relationship between functional genetic polymorphisms of *FGFR2* and variations in facial morphology.

**Methods:**

Frontal and lateral photographs of healthy boys (aged 9 to 14 years) and girls (aged 9 to 13 years), taken before the start of orthodontic treatment, were used for the study. Saliva samples from the patients were also analyzed. Patients with syndromes, skull trauma, or cleft lip and palate were excluded from the study. The measurements and determinations of proportions were performed using the Image J image processing program. DNA was extracted from the saliva samples to investigate seven genetic polymorphisms in *FGFR2* using real-time PCR (rs2162540, rs2981578, rs10736303, rs1078806, rs11200014, rs1219648, and rs4752566). ANOVA and t-tests were used to investigate the relationship between the genotypes and the horizontal as well as lateral facial proportions.

**Results:**

A total of 102 children (60 boys and 42 girls) were included in the analysis. No significant sex-related differences in relative facial dimensions or angles were observed. *FGFR2* polymorphisms were significantly associated with distinct facial parameters. The rs10736303 variant was associated with frontal facial proportions related to the eye and midface region, with larger relative dimensions observed in carriers of the AA genotype compared to the AG genotype. The rs2162540 polymorphism was associated with lateral profile measurements, including the relative vermilion height of the lower lip and the mentolabial angle. In addition, rs11200014 showed a significant association with the mentolabial angle, with larger angles observed in AA genotype carriers.

**Conclusion:**

This exploratory study suggested that *FGFR2* polymorphisms may be involved in specific facial parameters, supporting a role of *FGFR2* in facial development.

**Supplementary Information:**

The online version contains supplementary material available at 10.1186/s13005-026-00649-3.

## Introduction

Facial appearance represents a fundamental phenotypic attribute of human individuals, serving as a primary determinant of interpersonal recognition, social perception, and morphological identity. Facial structures formation result from the intricacy of morphogenesis involving many environmental and genetic factors. The role of genes in human facial formation has long been historically supported by evidence of familial similarities, especially for monozygotic twins [1]. Research aiming to unravel the critical genetic factors defining the size and shape of facial features has been developing rapidly in the last twenty years [2–9]. Several studies have explored the connection between genes involved in syndromes and cleft lip and palate with normal variations in the morphology of the face [1, 2, 4, 8].

Fibroblast growth factor receptor 2 (FGFR2) is a protein that plays an essential role in craniofacial development [10–12]. Mutations in the gene that encode this protein are related to several craniosynostosis syndromes. These syndromes arises from the premature fusion of cranial sutures, resulting in alterations to the craniofacial skeleton that manifest as complex and characteristic facial dysmorphisms, such as midface hypoplasia and orbital proptosis [13–17].

A variation in the DNA sequence that occurs in a population with a frequency of 1% or higher is termed a polymorphism [18]. A polymorphic variant of a gene can lead to variations in the expression or to the production of a different form of the protein, which may cause or be associated with some trait variations, such as facial traits [19]. Previous studies observed that genetic polymorphisms in *FGFR2* are involved in skeletal Class II and Class III malocclusions, which are dentofacial conditions related to abnormal positional or dimensional relationship discrepancies of the maxilla and mandible [20, 21]. Since the mutations in *FGFR2* have a big impact on the development of the face, and common genetic polymorphisms in *FGFR2* are involved in skeletal facial patterns, we expect that common polymorphisms in *FGFR2* are also involved in the normal variation of the facial morphology of humans. Therefore, in the present exploratory study we aimed to evaluate if genetic polymorphisms in *FGFR2* are associated with facial traits.

## Methods

The study was conducted and designed using Strengthening the Reporting of Genetic Association study (STREGA) statement checklist. Orthodontic patients were recruited from the Regensburg, Germany, between January 2020 and June 2021 with similar ethnicity, age, and sociocultural backgrounds.

This cross-sectional exploratory phenotype-genotype association study used facial photographs from orthodontic records to determine the phenotypes and DNA samples from all participants were analyzed to investigate genetic polymorphisms in *FGFR2*.

### Population and settings

A sample size calculation was performed based on the minor genotype frequency of the polymorphisms in *FGFR2* with the lowest frequency (0.15) and an expected facial measurement mean difference of 0.09 (standard deviation of 0.1) among genotypes. With an established alpha of 5% and power of 80% a minimum of 73 patients would be necessary. The data mean difference was based on a previously published study in another population [9].

Only patients with European ancestry (father, mother and their grandparents had European ancestry) were recruited. The patients’ ethnicity was self-declared. Only healthy children were included in the present study. Due to the fact that orthodontic treatment has an impact on facial appearance only patients before undergoing orthodontic treatment were included. Exclusion criteria were the presence of syndromes, cleft lip and/or palate and illnesses which would affect the facial appearance. Patients with tooth agenesis (excluding third molars) were excluded. Furthermore, patients with missing or bad quality photographs or saliva samples were excluded.

### Phenotype determination: landmarks and measurements

Existing digital frontal and lateral photographs, which were taken before undergoing orthodontic therapy, were used to investigate and measure facial parameters.

**Fig. 1:**
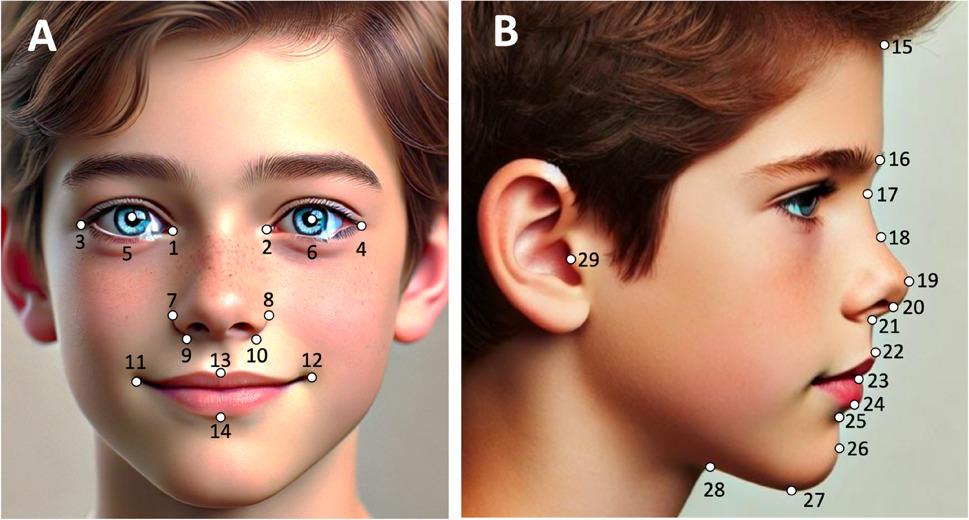
Soft tissue landmarks used. (**A**) Frontal image. (**B**) Lateral image

The main criteria for determining the measuring points were, in particular, good reproducibility and visibility [22]. Following the protocol of recently published studies [22–24] 29 (unilateral and bilateral) landmarks were used (Fig. 1). The used frontal soft tissue landmarks are shown in Fig. 1A and lateral soft tissue landmarks are shown in Fig. 1B. The definitions of the landmarks are presented in Table 1.

**Table 1:** Definition of facial landmarks

No.	Landmark	Definition
1–2	Inner canthus (iCthr-iCthl)	The soft tissue point located at the inner commissure of each eye fissure
3–4	Outer canthus (oCthr-oCthl)	The soft tissue point located at the outer commissure of each eye fissure
5–6	Pupil (Pupr-Pupl)	The center point of the eye pupil
7–8	Alar (Alr-All)	The most lateral point of the alar contour of the nose
9–10	Alar base (Albr-Albl)	The point on the lower margin of the alar base where the alar disappears into the upper lip skin
11–12	Lip commissure (Lcr-Lcl)	The point where the lips join together at the side of the mouth
13–14	Lip superior and inferior (Ls-Li)	The point that indicates the mucocutaneous limit of the upper and lower lip
15	Trichion (Tri)	The sagittal midpoint of the forehead that borders the hairline
16	Glabella (G)	The most anterior point of the middle line of the forehead
17	Nasion (N)	The point in the middle line located at the nasal root
18	Midnasal (Mn)	The middle point on the outer contour of the nose between the pronasal and nasion points
19	Pronasal (Prn)	The most prominent point of the tip of the nose
20	Columnella (Cm)	The most inferior and anterior point of the nose
21	Subnasal (Sn)	The point where the upper lip joins the columella
22	Labial superior (Ls)	The point that indicates the mucocutaneous limit of the upper lip
23	Stomion (Sts = Sti)	The midpoint of the labial fissure when the lips are closed naturally
24	Labial inferior (Li)	The point that indicates the mucocutaneous limit of the lower lip
25	Supramental (Sm)	The deepest point of the inferior sublabial concavity
26	Progonion (Pg)	The most anterior point of the chin
27	Menton (Me)	The most inferior point of the inferior edge of the chin
28	Cervical (C)	The point at the junction of neck and throat borders
29	Tragus (Trg)	The most posterior point of the auricular tragus

The images were adapted from an AI-generated image created using ChatGPT (January 2025, version 4), OpenAI.

The used reference lines in this study were the bipupillary line (goes through Pupr-Pupl) for frontal pictures. For lateral pictures the used reference line was the Frankfurter horizontal line, which extends from the lowest point at the lower edge of the orbit to the highest point at the upper edge of the external auditory canal (Meatus acusticus externus), the porion [25].

On the basis of these landmarks, we performed 22 measurements per patient described in Table 2. The localization of landmarks and the measurements were performed by a single calibrated evaluator. Briefly, 10 frontal and 10 profile pictures were used for concordance assessment. The examiner repeated detection and the measurements at a 2 weeks’ interval. The Intraclass Correlation Coefficient (ICC) was calculated and ranged from 0.93 to 0.99.

**Table 2:** Linear and angular measurements evaluated in this study

Measurement	Description	Reference
Frontal		
a	Inner intercanthal distance (iCthr-iCthl)	Moshkelgosha et al., 2015; Ozdemir et al., 2009
b	Outer intercanthal distance (oCthr-oCthl)	Moshkelgosha et al., 2015; Ozdemir et al., 2009
c	Pupil midface distance (Pupr-Pupl)	Moshkelgosha et al., 2015; Ozdemir et al., 2009
d	Alar width (Alr-All)	Moshkelgosha et al., 2015; Ozdemir et al., 2009
e	Nasal base width (Albr-Albl)	Moshkelgosha et al., 2015; Ozdemir et al., 2009
f	Mouth width (Lcr-Lcl)	Moshkelgosha et al., 2015; Ozdemir et al., 2009
g	Lip height (Ls-Li)	Moshkelgosha et al., 2015; Ozdemir et al., 2009
Profile Linear		
h	Superior facial third (Tri-G)	Fernández-Riveiro et al., 2002; Speckmann et al., 2004; Moshkelgosha et al., 2015
i	Middle facial third (G-Sn)	Fernández-Riveiro et al., 2002; Speckmann et al., 2004; Moshkelgosha et al., 2015
j	Inferior facial third (Sn-Me)	Fernández-Riveiro et al., 2002; Speckmann et al., 2004; Moshkelgosha et al., 2015
k	Length of upper lip (Sn-Sts)	Fernández-Riveiro et al., 2002; Speckmann et al., 2004; Moshkelgosha et al., 2015
l	Length of lower lip (Sti-Sm)	Fernández-Riveiro et al., 2002; Speckmann et al., 2004; Moshkelgosha et al., 2015
m	Vermilion of upper lip (Ls-Sts)	Fernández-Riveiro et al., 2002; Speckmann et al., 2004; Moshkelgosha et al., 2015
n	Vermilion of lower lip (Li-Sti)	Fernández-Riveiro et al., 2002; Speckmann et al., 2004; Moshkelgosha et al., 2015
o	Height of chin (Sm-Me)	Fernández-Riveiro et al., 2002; Speckmann et al., 2004; Moshkelgosha et al., 2015
Profile Angular		
16-17-19	Nasofrontal angle (G-N-Prn)	Fernández-Riveiro et al., 2003; Speckmann et al., 2004; Moshkelgosha et al., 2015
20-21-22	Nasolabial angle (Cm–Sn–Ls)	Fernández-Riveiro et al., 2003; Speckmann et al., 2004; Moshkelgosha et al., 2015
24-25-26	Mentolabial angle (Li–Sm–Pg)	Fernández-Riveiro et al., 2003; Speckmann et al., 2004; Moshkelgosha et al., 2015
17-29-21	Angle of the medium facial third (N–Trg–Sn)	Fernández-Riveiro et al., 2003; Speckmann et al., 2004; Moshkelgosha et al., 2015
21-29-27	Angle of the inferior facial third (Sn–Trg–Me)	Fernández-Riveiro et al., 2003; Speckmann et al., 2004; Moshkelgosha et al., 2015
21 − 20/17–19	Nasal angle (Sn–Cm/N–Prn)	Fernández-Riveiro et al., 2003; Speckmann et al., 2004; Moshkelgosha et al., 2015
17-18-19	Angle of the nasal dorsum (N–Mn–Prn)	Fernández-Riveiro et al., 2003; Speckmann et al., 2004; Moshkelgosha et al., 2015
16–26/28 − 27	Cervicomental angle (G–Pg/C–Me)	Fernández-Riveiro et al., 2003; Speckmann et al., 2004; Moshkelgosha et al., 2015
16-21-26	Angle of facial convexity (G–Sn–Pg)	Fernández-Riveiro et al., 2003; Speckmann et al., 2004; Moshkelgosha et al., 2015
16-19-26	Angle of total facial convexity (G–Prn–Pg)	Fernández-Riveiro et al., 2003; Speckmann et al., 2004; Moshkelgosha et al., 2015

**Fig. 2:**
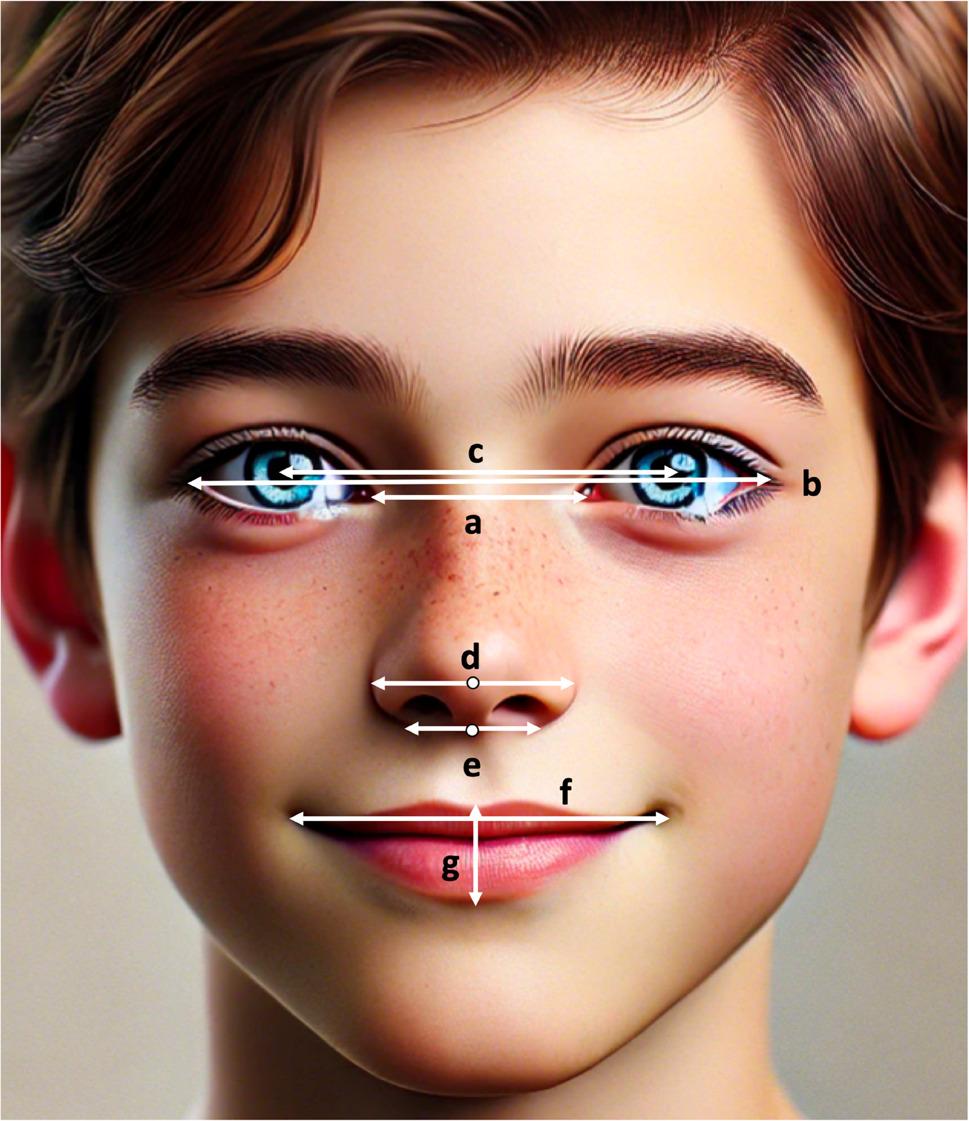
Shows the measurements performed on frontal images. Frontal image measurements. The image was adapted from an AI-generated image created using ChatGPT (January 2025, version 4), OpenAI

**Fig. 3:**
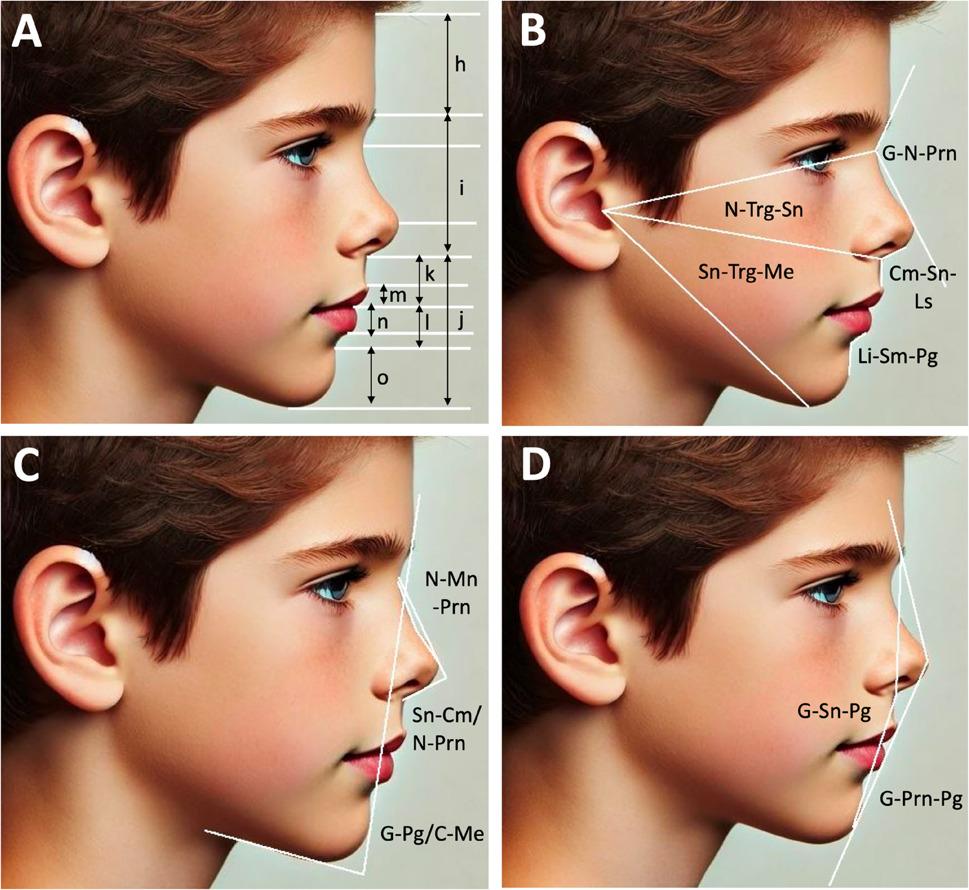
(**A**, **B**, **C** and **D**) shows the measurements performed on frontal images. Lateral image measurements. (**A**) Linear measurements; (**B**–**D**) Angular measurements. The images were adapted from an AI-generated image created using ChatGPT (January 2025, version 4), OpenAI

The software used in our study was ImageJ (National Institutes of Health, Bethesda, MD, USA). Frontal and lateral images (Figs. 2 and 3) were aligned according to the reference lines. The photographs were taken without a fixed size scale, therefore only relative values such as ratios and angles were calculated [26]. The software was used to record the absolute distance as the number of pixels and subsequently displayed as a relation.

On frontal images, the relationships between the following measurements were calculated (right = r; left = l):

- Inner intercanthal distance (iCthr-iCthl) and outer intercanthal distance (oCthr-oCthl) [iCthr-iCthl: oCthr-oCthl].
- Inner intercanthal distance (iCthr-iCthl) and Pupil midface distance (Pupr-Pupl) [iCthr-iCthl: Pupr-Pupl].
- Nasal base width (Albr-Albl) and Alar width (Alr-All) [Albr-Albl: Alr-All].
- Lip height (Ls-Li) and Mouth width (Lcr-Lcl) [Ls-Li: Lcr-Lcl].

On lateral images, the parameters were calculated by the following formulas:

- Superior facial third (Tri-G) [Tri-G: Tri-Mn].
- Middle facial third (G-Sn) [G-Sn: Tri-Mn].
- Inferior facial third (Sn-Me) [Sn-Me: Tri-Mn].
- Length of upper lip (Sn-Sts) [Sn-Sts: Sn-Me].
- Length of lower lip (Sti-Sm) [Sti-Sm: Sn-Me].
- Vermilion of upper lip (Ls-Sts) [Ls-Sts: Sn-Me].
- Vermilion of lower lip (Li-Sti) [Li-Sti: Sn-Me].
- Height of chin (Sm-Me) [Sm-Me: Sn-Me].

### Genotyping

The genetic polymorphisms in *FGFR2* were selected based on their minor allele frequency that should be higher than 20%. The description of the selected polymorphisms is presented in Table 3.

**Table 3:** Characteristics of the investigated polymorphisms in FGFR2

Chromosome	rs	Base change	MAF	Description	Genotype success rate
10:121592622	rs2162540	C > T	0.433	Located on Intron 2; Skeletal Facial morphology–associated	98/102
10:121580797	rs2981578	C > T	0.394	Located on Intron 2; Skeletal Facial morphology–associated	88/102
10:121574943	rs10736303	A > G	0.392	Located on Intron 2; Skeletal Facial morphology–associated	94/102
10:121579461	rs1078806	A > G	0.340	Located on Intron 2	94/102
10:121575416	rs11200014	A > G	0.341	Located on Intron 2; Skeletal Facial morphology–associated	89/102
10:121586676	rs1219648	A > G	0.395	Located on Intron 2 variant	94/102
10:121508117	rs4752566	G > T	0.374	Located on Intron 9 variant	92/102

Data also obtained from databases: http://www.thermofisher.com; https://www.ncbi.nlm.nih.gov/snp. MAF means Minor Allele Frequency

Genomic DNA extracted from cells isolated from saliva samples [27] was used for the genotyping analysis. The assessment of DNA quantity and purity was conducted using spectrophotometry (Nanodrop 1000; Thermo Scientific). The polymorphisms were genotyped blindly via real-time polymerase chain reaction (PCR) utilizing the TaqMan assay (Step One Plus Real-Time PCR System, Applied Biosystems, Foster City, CA).

### Statistical analysis

The dependent variables (Facial measurements) were evaluated as continuous variables. The independent variables were assessed as categorical variables for each genetic polymorphism.

Regarding the genotyping data, Hardy-Weinberg equilibrium was tested by chi-square test (https://wpcalc.com/en/equilibrium-hardy-weinberg/) before further analysis. A one-way ANOVA was performed to analyze the means distribution among genotypes (α = 5%). All statistical analyses were performed using the Statistical Package for Social Sciences 20.0 software (SPSS, Chicago, IL, USA).

## Results

Orthodontic records including pictures and saliva samples were screened and 102 healthy children (60 males, 42 females) were included in the study. They aged 9–14 (males) and 9–13 (females). The different age ranges result from the fact that female patients experience a growth spurt about one year earlier than boys. Relative facial dimensions and facial angles were not statistically significant among genders and are shown in Table 4.

**Table 4:** P-values for dimension comparisons among genders

Measurements	*p*-value
iCthr-iCthl : oCthr-oCthl	0.811
iCthr-iCthl : Pupr-Pupl	0.159
Albr-Albl : Alr-All	0.542
Ls-Li : Lcr-Lcl	0.648
Tri-G relative	0.139
G-Sn relative	0.346
Sn-Me relative	0.392
Sn-Sts relative	0.900
Sti-Sm relative	0.121
Ls-Sts relative	0.546
Li-Sti relative	0.537
Sm-Me relative	0.375
G-N–Prn (nasofrontal angle )	0.534
Cm–Sn–Ls (nasolabial angle)	0.053
Li–Sm–Pg (mentolabial angle)	0.774
Sn–Cm/N–Prn (nasal angle )	0.777
N–Mn–Prn (angle of the nasal dorsum)	0.224
G–Pg/C–Me (cervicomental angle)	0.057
N–Trg–Sn (angle of the medium facial third)	0.329
Sn–Trg–Me (angle of the inferior facial third)	0.893
G–Sn–Pg (angle of facial convexity)	0.107
G–Prn–Pg (angle of total facial convexity)	0.363

All genetic polymorphisms were within the HWE (χ2value for rs2162540 = 3.27; χ2value for rs2981578 = 0.42; χ2value for rs10736303 = 1.02; χ2value for rs1078806 = 0.18; χ2value for rs11200014 = 0.06; χ2value for rs1219648 = 0.64; χ2value for rs4752566 = 0.01). The genotype success rate ranged from 86.3% to 96.1% and is presented in Table 3.

The frontal face relative dimensions according to genotypes in *FGFR2* are presented in Table 5. The polymorphism rs10736303 was associated with relative dimension related to proportion of the eyes and midface. The relative dimension of the inner intercanthal distance to the outer intercanthal distance (iCthr-iCthl: oCthr-oCthl) was associated with the rs10736303 (*p* = 0.013). The relative dimension of the inner intercanthal distance and Pupil midface distance (iCthr-iCthl: Pupr-Pupl) was associated with the rs10736303 (*p* = 0.026). Patients carrying the AA genotype had larger dimensions than those carrying the AG genotype.

**Table 5:** Frontal face proportions according to FGFR2 genotypes

Measurements	Mean (SD)			*p*-values
rs2162540	CC (*n* = 12)	CT (*n* = 56)	TT (*n* = 30)	
iCthr-iCthl : oCthr-oCthl	0.381 (0.024)	0.380 (0.028)	0.381 (0.017)	0.964
iCthr-iCthl : Pupr-Pupl	0.537 (0.025)	0.536 (0.034)	0.536 (0.021)	0.996
Albr-Albl : Alr-All	0.693 (0.055)	0.676 (0.051)	0.669 (0.063)	0.456
Ls-Li : Lcr-Lcl	0.283 (0.054)	0.311 (0.072)	0.294 (0.058)	0.290
rs2981578	CC (*n* = 22)	CT (*n* = 47)	TT (*n* = 19)	
iCthr-iCthl : oCthr-oCthl	0.373 (0.023)	0.379 (0.025)	0.385 (0.022)	0.272
iCthr-iCthl : Pupr-Pupl	0.525 (0.028)	0.535 (0.031)	0.540 (0.026)	0.220
Albr-Albl : Alr-All	0.676 (0.060)	0.678 (0.052)	0.662 (0.064)	0.567
Ls-Li : Lcr-Lcl	0.297 (0.054)	0.313 (0.076)	0.295 (0.044)	0.460
rs10736303	AA (*n* = 31)	AG (*n* = 50)	GG (*n* = 13)	
iCthr-iCthl : oCthr-oCthl	0.389 (0.022)^a^	0.374 (0.022)^b^	0.384 (0.029)^a, b^	0.013*
iCthr-iCthl : Pupr-Pupl	0.547 (0.026) ^a^	0.529 (0.029) ^b^	0.538 (0.033) ^a, b^	0.026*
Albr-Albl : Alr-All	0.661 (0.055)	0.676 (0.056)	0.704 (0.040)	0.064
Ls-Li : Lcr-Lcl	0.300 (0.067)	0.304 (0.067)	0.305 (0.068)	0.965
rs1078806	AA (*n* = 38)	AG (*n* = 42)	GG (*n* = 14)	
iCthr-iCthl : oCthr-oCthl	0.386 (0.022)	0.376 (0.025)	0.380 (0.024)	0.144
iCthr-iCthl : Pupr-Pupl	0.543 (0.026)	0.531 (0.032)	0.531 (0.025)	0.159
Albr-Albl : Alr-All	0.660 (0.055)	0.686 (0.055)	0.688 (0.054)	0.077
Ls-Li : Lcr-Lcl	0.303 (0.064)	0.300 (0.076)	0.301 (0.051)	0.977
rs11200014	AA (*n* = 11)	AG (*n* = 39)	GG (*n* = 39)	
iCthr-iCthl : oCthr-oCthl	0.379 (0.023)	0.374 (0.021)	0.386 (0.024)	0.069
iCthr-iCthl : Pupr-Pupl	0.534 (0.025)	0.528 (0.028)	0.542 (0.029)	0.100
Albr-Albl : Alr-All	0.702 (0.052)	0.681(0.051)	0.661 (0.054)	0.054
Ls-Li : Lcr-Lcl	0.277 (0.052)	0.307 (0.073)	0.306 (0.066)	0.412
rs1219648	AA (*n* = 42)	AG (*n* = 39)	GG (*n* = 13)	
iCthr-iCthl : oCthr-oCthl	0.386 (0.023)	0.374 (0.024)	0.380 (0.024)	0.053
iCthr-iCthl : Pupr-Pupl	0.543 (0.027)	0.528 (0.032)	0.536 (0.025)	0.076
Albr-Albl : Alr-All	0.661 (0.054)	0.681 (0.055)	0.695 (0.052)	0.085
Ls-Li : Lcr-Lcl	0.301 (0.065)	0.307 (0.073)	0.292 (0.054)	0.768
rs4752566	GG (*n* = 32)	GT (*n* = 45)	TT (*n* = 15)	
iCthr-iCthl : oCthr-oCthl	0.384 (0.029)	0.377 (0.021)	0.380 (0.016)	0.404
iCthr-iCthl : Pupr-Pupl	0.541 (0.034	0.532 (0.027)	0.533 (0.022)	0.359
Albr-Albl : Alr-All	0.682 (0.055)	0.676 (0.053)	0.655 (0.063)	0.287
Ls-Li : Lcr-Lcl	0.308 (0.081)	0.292 (0.057)	0.309 (0.061)	0.488

*indicates *p* < 0.05. Means with the same letter are not significantly different from each other

Table 6 shows the lateral profile proportions according to genotypes in *FGFR2*. An association was observed for the polymorphism rs2162540 for the measurement Vermilion of lower lip relative to inferior facial third (Li-Sti relative), in which patients carrying the genotype CC had smaller vermilion of lower lip proportion in relation to the inferior facial third than patients carrying CT genotype (*p* = 0.049).

**Table 6:** Lateral profile proportions according to FGFR2 genotypes

Measurements	Mean (SD)			*p*-values
rs2162540	CC (*n* = 12)	CT (*n* = 56)	TT (*n* = 30)	
Tri-G	0.307 (0.025)	0.318 (0.027)	0.314 (0.020)	0.406
G-Sn	0.344 (0.023)	0.341 (0.021)	0.343 (0.020)	0.809
Sn-Me	0.349 (0.020)	0.342 (0.020)	0.343 (0.019)	0.540
Sn-Sts	0.331 (0.028)	0.322 (0.030)	0.333 (0.035)	0.237
Sti-Sm	0.245 (0.032)	0.244 (0.023)	0.241 (0.034)	0.869
Ls-Sts	0.118 (0.018)	0.129 (0.024)	0.122 (0.023)	0.185
Li-Sti	0.126 (0.018)^a^	0.144 (0.026)^b^	0.132 (0.029)^a, b^	0.049*
Sm-Me	0.425 (0.042)	0.434 (0.033)	0.427 (0.038)	0.556
rs2981578	CC (*n* = 22)	CT (*n* = 47)	TT (*n* = 19)	
Tri-G	0.313 (0.022)	0.316 (0.029)	0.314 (0.026)	0.883
G-Sn	0.343 (0.020)	0.341 (0.022)	0.342 (0.019)	0.906
Sn-Me	0.344 (0.018)	0.343 (0.020)	0.344 (0.020)	0.976
Sn-Sts	0.328 (0.025)	0.325 (0.032)	0.331 (0.037)	0.779
Sti-Sm	0.245 (0.027)	0.247 (0.024)	0.242 (0.035)	0.841
Ls-Sts	0.124 (0.021)	0.130 (0.022)	0.121 (0.025)	0.228
Li-Sti	0.140 (0.021)	0.142 (0.026)	0.135 (0.026)	0.571
Sm-Me	0.425 (0.037)	0.431 (0.034)	0.430 (0.034)	0.800
rs10736303	AA (*n* = 31)	AG (*n* = 50)	GG (*n* = 13)	
Tri-G	0.317 (0.028)	0.312 (0.022)	0.323 (0.031)	0.381
G-Sn	0.342 (0.021)	0.343 (0.019)	0.334 (0.025)	0.312
Sn-Me	0.341(0.022)	0.345 (0.018)	0.344 (0.019)	0.648
Sn-Sts	0.326 (0.035)	0.326 (0.032)	0.332 (0.024)	0.844
Sti-Sm	0.241 (0.033)	0.242 (0.026)	0.250 (0.022)	0.583
Ls-Sts	0.126 (0.024)	0.127 (0.024)	0.124 (0.014)	0.874
Li-Sti	0.137 (0.029)	0.137 (0.027)	0.144 (0.022)	0.732
Sm-Me	0.434 (0.038)	0.432 (0.034)	0.428 (0.038)	0.898
rs1078806	AA (*n* = 38)	AG (*n* = 42)	GG (*n* = 14)	
Tri-G	0.317 (0.027)	0.313 (0.025)	0.315 (0.023)	0.778
G-Sn	0.343 (0.020)	0.342 (0.021)	0.340 (0.023)	0.896
Sn-Me	0.340 (0.021)	0.345 (0.019)	0.345 (0.019)	0.485
Sn-Sts	0.330 (0.034)	0.323 (0.025)	0.335 (0.039)	0.351
Sti-Sm	0.242 (0.032)	0.243 (0.027)	0.244 (0.021)	0.957
Ls-Sts	0.125 (0.024)	0.128 (0.025)	0.121 (0.016)	0.655
Li-Sti	0.138 (0.029)	0.137 (0.028)	0.135 (0.018)	0.910
Sm-Me	0.429 (0.038)	0.436 (0.033)	0.423 (0.037)	0.473
rs11200014	AA (*n* = 11)	AG (*n* = 39)	GG (*n* = 39)	
Tri-G	0.308 (0.026)	0.312 (0.025)	0.319 (0.027)	0.333
G-Sn	0.343 (0.022)	0.341 (0.021)	0.341 (0.021)	0.942
Sn-Me	0.349 (0.017)	0.347 (0.019)	0.339 (0.021)	0.156
Sn-Sts	0.327 (0.029)	0.325 (0.030)	0.329 (0.035)	0.823
Sti-Sm	0.247 (0.028)	0.240 (0.025)	0.241 (0.031)	0.753
Ls-Sts	0.115 (0.019)	0.130 (0.023)	0.126 (0.024)	0.200
Li-Sti	0.130 (0.017)	0.137 (0.028)	0.141 (0.029)	0.509
Sm-Me	0.424 (0.043)	0.438 (0.031)	0.430 (0.036)	0.451
rs1219648	AA (*n* = 42)	AG (*n* = 39)	GG (*n* = 13)	
Tri-G	0.317 (0.026)	0.316 (0.025)	0.305 (0.025)	0.317
G-Sn	0.344 (0.021)	0.339 (0.020)	0.342 (0.019)	0.515
Sn-Me	0.338 (0.021)	0.346 (0.019)	0.352 (0.016)	0.053
Sn-Sts	0.328 (0.034)	0.325 (0.030)	0.331 (0.027)	0.855
Sti-Sm	0.243 (0.031)	0.242 (0.024)	0.245 (0.031)	0.941
Ls-Sts	0.124 (0.023)	0.130 (0.024)	0.122 (0.022)	0.355
Li-Sti	0.139 (0.029)	0.139 (0.027)	0.131 (0.019)	0.594
Sm-Me	0.430 (0.037)	0.435 (0.031)	0.426 (0.041)	0.674
rs4752566	GG (*n* = 32)	GT (*n* = 45)	TT (*n* = 15)	
Tri-G	0.314 (0.028)	0.313 (0.025)	0.326 (0.018)	0.207
G-Sn	0.340 (0.019)	0.344 (0.022)	0.339 (0.020)	0.565
Sn-Me	0.347 (0.020)	0.343 (0.020)	0.335 (0.020)	0.179
Sn-Sts	0.327 (0.030)	0.329 (0.029)	0.327 (0.032)	0.939
Sti-Sm	0.241 (0.025)	0.240 (0.029)	0.248 (0.030)	0.616
Ls-Sts	0.132 (0.027)	0.123 (0.020)	0.122 (0.026)	0.230
Li-Sti	0.140 (0.032)	0.133 (0.025)	0.143 (0.023)	0.414
Sm-Me	0.432 (0.032)	0.433 (0.036)	0.428 (0.038)	0.885

* indicates *p* < 0.05. Means with the same letter are not significantly different from each other

Table 7 shows the facial angles according to genotypes in *FGFR2*. The mentolabial angle (Li–Sm–Pg) showed an association (*p* = 0.044), in which patients carrying CC for the polymorphism rs2162540 had a bigger angle than patients carrying CT genotype. The polymorphism rs11200014 was also associated with the mentolabial angle Li–Sm–Pg, patients carrying AA genotype had bigger angle than patients carrying AG and GG genotypes (*p* = 0.024).

**Table 7:** Facial angles according to FGFR2 genotypes

Angles	Mean (SD)			*p*-value
rs2162540	CC (*n* = 12)	CT (*n* = 56)	TT (*n* = 30)	
G-N–Prn	138.853 (5.576)	140.885 (5.569)	140.202 (4.949)	0.480
Cm–Sn–Ls	116.601 (7.874)	116.655 (9.606)	114.844 (9.281)	0.679
Li–Sm–Pg	135.963 (12.165)^a^	126.133 (13.552)^b^	130.152 (11.679) ^a, b^	0.044*
Sn–Cm/N–Prn	91.207 (5.672)	92.325 (6.781)	93.144 (7.779)	0.708
N–Mn–Prn	183.557 (4.941)	183.463 (5.867)	185.273 (7.190)	0.422
G–Pg/C–Me	100.055 (8.214)	102.466 (8.407)	101.593 (8.116)	0.640
N–Trg–Sn	27.117 (2.225)	26.848 (2.261)	27.282 (2.108)	0.677
Sn–Trg–Me	37.828 (2.713)	36.991 (2.513)	37.318 (2.657)	0.567
G–Sn–Pg	166.969 (5.441)	165.558 (5.011)	166.310 (3.578)	0.567
G–Prn–Pg	141.395 (4.416)	140.511 (5.132)	141.966 (4.966)	0.428
rs2981578	CC (*n* = 22)	CT (*n* = 47)	TT (*n* = 19)	
G-N–Prn	139.013 (5.745)	140.460 (5.837)	141.556 (4.261)	0.335
Cm–Sn–Ls	117.635 (8.831)	115.986 (9.978)	116.208 (8.664)	0.790
Li–Sm–Pg	129.946 (16.595)	126.556 (10.417)	131.006 (14.398)	0.372
Sn–Cm/N–Prn	92.665 (6.463)	91.862 (7.642)	93.380 (6.578)	0.722
N–Mn–Prn	182.543 (4.871)	184.844 (6.762)	184.971 (7.836)	0.359
G–Pg/C–Me	102.442 (8.792)	101.946 (8.099)	102.667 (8.673)	0.942
N–Trg–Sn	26.964 (1.939)	27.011 (2.236)	27.390 (2.484)	0.789
Sn–Trg–Me	37.622 (2.642)	37.270 (2.503)	37.277 (2.815)	0.862
G–Sn–Pg	165.539 (4.585)	165.414 (4.997)	167.221 (3.011)	0.327
G–Prn–Pg	140.567 (4.172)	140.419 (5.589)	143.106 (4.385)	0.134
rs10736303	AA (*n* = 31)	AG (*n* = 50)	GG (*n* = 13)	
G-N–Prn	140.827 (4.443)	140.767 (5.655)	138.855 (6.166)	0.484
Cm–Sn–Ls	116.801 (9.145)	115.841 (9.187)	115.336 (10.804)	0.863
Li–Sm–Pg	128.805 (12.395)	128.377 (12.640)	128.238 (15.921)	0.987
Sn–Cm/N–Prn	93.427 (6.338)	92.017 (6.982)	90.742 (8.396)	0.467
N–Mn–Prn	185.644 (6.736)	183.916 (6.082)	181.395 (5.111)	0.114
G–Pg/C–Me	101.888 (8.681)	102.465 (7.841)	98.648 (9.382)	0.341
N–Trg–Sn	27.434 (2.321)	27.021 (2.128)	26.051 (2.078)	0.166
Sn–Trg–Me	37.288 (2.645)	37.306 (2.547)	36.688 (1.914)	0.717
G–Sn–Pg	166.145 (4.379)	166.140 (4.870)	164.979 (5.219)	0.717
G–Prn–Pg	141.845 (5.328)	140.965 (5.054)	139.786 (4.623)	0.461
rs1078806	AA (*n* = 38)	AG (*n* = 42)	GG (*n* = 14)	
G-N–Prn	140.747 (4.781)	140.279 (5.780)	140.755 (5.824)	0.916
Cm–Sn–Ls	116.435 (9.076)	115.527(9.701)	116.934 (9.142)	0.854
Li–Sm–Pg	128.116 (12.273)	128.917 (14.265)	131.944 (11.111)	0.643
Sn–Cm/N–Prn	93.337 (6.610)	90.748 (7.406)	94.447 (5.574)	0.117
N–Mn–Prn	185.593 (6.691)	182.615 (6.046)	184.117 (5.170)	0.106
G–Pg/C–Me	100.942 (8.124)	102.402 (8.025)	102.008 (10.293)	0.736
N–Trg–Sn	27.360 (2.337)	26.845 (2.231)	26.516 (1.755)	0.395
Sn–Trg–Me	37.454 (2.633)	36.788 (2.362)	37.865 (2.364)	0.282
G–Sn–Pg	165.802 (4.136)	166.444 (5.305)	164.695 (4.076)	0.475
G–Prn–Pg	141.241 (5.536)	141.169 (4.990)	140.089 (4.303)	0.754
rs11200014	AA (*n* = 11)	AG (*n* = 39)	GG (*n* = 39)	
G-N–Prn	139.364 (6.209)	140.770 (5.908)	141.005 (4.665)	0.673
Cm–Sn–Ls	120.728 (7.596)	115.095 (9.482)	116.477 (9.247)	0.204
Li–Sm–Pg	138.303 (11.583)^a^	126.624 (12.743)^b^	127.837 (12.334)^b^	0.024*
Sn–Cm/N–Prn	93.205 (7.398)	91.699 (7.479)	92.657 (6.679)	0.759
N–Mn–Prn	182.460 (4.979)	183.472 (6.566)	184.998 (6.416)	0.393
G–Pg/C–Me	101.095 (7.278)	102.860 (8.335)	101.514 (8.744)	0.719
N–Trg–Sn	27.326 (2.134)	26.542 (2.175)	27.410 (2.290)	0.206
Sn–Trg–Me	37.893 (2.644)	37.073 (2.072)	37.418 (2.806)	0.600
G–Sn–Pg	165.863 (5.395)	166.392 (4.792)	165.175 (4.550)	0.531
G–Prn–Pg	140.496 (3.917)	141.373 (5.283)	140.590 (5.370)	0.768
rs1219648	AA (*n* = 42)	AG (*n* = 39)	GG (*n* = 13)	
G-N–Prn	140.814 (4.642)	140.499 (5.874)	139.859 (6.169)	0.854
Cm–Sn–Ls	116.790 (9.014)	115.230 (9.864)	117.759 (8.609)	0.624
Li–Sm–Pg	127.848 (12.335)	127.089 (13.314)	134.929 (12.489)	0.150
Sn–Cm/N–Prn	92.922 (6.537)	91.562 (7.429)	92.946 (7.012)	0.648
N–Mn–Prn	184.947 (6.723)	183.369 (6.219)	183.761 (4.942)	0.520
G–Pg/C–Me	101.328 (7.915)	103.078 (8.992)	99.340 (7.663)	0.341
N–Trg–Sn	27.289 (2.071)	26.566 (2.078)	27.520 (2.853)	0.231
Sn–Trg–Me	37.183 (2.713)	37.065 (2.286)	37.698 (2.559)	0.734
G–Sn–Pg	165.451 (4.231)	166.059 (5.001)	166.853 (5.231)	0.620
G–Prn–Pg	140.714 (5.418)	141.104 (5.081)	141.910 (4.189)	0.760
rs4752566	GG (*n* = 32)	GT (*n* = 45)	TT (*n* = 15)	
G-N–Prn	139.270 (5.053)	141.444 (4.941)	140.271 (6.446)	0.203
Cm–Sn–Ls	116.678 (9.669)	116.061 (8.146)	115.288 (10.972)	0.886
Li–Sm–Pg	129.555 (11.919)	128.792 (15.127)	126.605 (9.671)	0.777
Sn–Cm/N–Prn	92.848 (6.329)	91.630 (6.344)	91.555 (8.801)	0.707
N–Mn–Prn	183.654 (5.622)	184.080 (6.180)	184.101 (8.350)	0.953
G–Pg/C–Me	100.357 (9.137)	103.098 (7.403)	100.780 (7.460)	0.301
N–Trg–Sn	26.535 (1.868)	27.239 (2.371)	27.297 (2.533)	0.344
Sn–Trg–Me	37.168 (2.680)	37.008 (2.148)	37.098 (3.436)	0.964
G–Sn–Pg	164.741 (4.609)	166.543 (4.878)	166.360 (4.383)	0.238
G–Prn–Pg	139.984 (4.673)	141.699 (5.518)	140.233 (4.186)	0.299

*indicates statistical differences. Means with the same letter are not significantly different from each other

## Discussion

Facial morphology is a well-known highly heritable and complex polygenic trait. It exhibits substantial phenotypic variation within human populations, reflecting the subtle yet cumulative effects of numerous genetic loci. The exact dimension, morphology, and spatial relationships of the underlying craniofacial skeleton and associated soft tissues are primarily governed by the intricate interplay of developmental pathways, notably those mediated by the FGF signaling cascades. Furthermore, loss- or gain-of-function mutations in these same critical developmental genes are responsible for severe deviations from typical morphology, leading to syndromic conditions like craniosynostoses [13–17], while common polymorphisms are associate with common dentofacial phenotypes, such as skeletal malocclusions [20, 21, 28], tooth agenesis [27] and dental root alteration [29].

Although the results of this exploratory study bring some interesting new information about the role of FGFR2 on facial development, one of the key limitations of the study is that a two-dimensional facial analysis was conducted exclusively using photographs, which restricted the assessment to proportional measurements rather than precise linear dimensions in millimetres. Also, the absence of three-dimensional measurements prevents a more robust evaluation of absolute facial dimensions. Linear and angular soft tissue facial analysis based on photogrammetry has been extensively described [23, 24, 30–34] and used in genetic studies of facial morphology [9], in which proportional assessments provide valuable comparative insights. However, they cannot fully substitute for standardized, millimeter-based measurements that would offer greater precision and diagnostic reliability. Another primary limitation is the absence of radiographic screening to confirm that the included children had not yet reached their peak growth spurt.

Another limitation that should be discussed here is the multiple comparisons performed. In our genetic association study, we analyzed 22 distinct phenotypic measurements, thus it is important to discuss the risk of a Type II error, or false negative, which could be critically exacerbated by the requirement for multiple testing correction. On the other hand, to adjust the nominal significance threshold (alpha) for the multiple comparisons would be extremely strict, leading to a severe reduction in the p-values cutoff and a reduction in the statistical power of the study for each individual measurement, facing a heightened risk of failing to detect a true genetic association. Particularly those polymorphisms with small to moderate effect sizes, even if the association genuinely exists, thereby increasing the likelihood of committing a Type II error. Therefore, the current study should be considered exploratory and future studies should be performed to confirm the current result.

The results support the possible role of FGFR2 in facial morphology. A previous study showed that *FGFR1* polymorphisms had an important role in the normal human skull variation, influencing the head length, which affects other cephalometric measurements [4]. In our study, the polymorphism rs10736303 in *FGFR2* was identified as a candidate for relative dimension related to proportion of the eyes and midface. Interestingly, mutations in the *FGFR2* gene are associated with syndromes like Crouzon, Apert, or Pfeiffer, which lead to the premature fusion of cranial and facial sutures, resulting in midface hypoplasia.

Another interesting possible association was observed for the polymorphism rs2162540 for the Vermilion of lower lip that was smaller in patients carrying genotype CC. The mentolabial angle also showed a statistical significance, in which patients carrying CC for the polymorphism rs2162540 had a bigger angle. The polymorphism rs11200014 was associated with the mentolabial angle, in which patients carrying AA genotype had bigger angle. These soft tissue phenotypes are common characteristics of patients with skeletal class II malocclusion. In the past years, genetic polymorphisms in *FGFR2* have been associated with skeletal malocclusions class II and III and with mandibular retrognathism [20, 21, 28]. Therefore, it is possible that the lip phenotypes observed here are the result of FGFR2 influencing the mandibular bone phenotype, or that FGFR2 also has an important role in lip formation.

While this exploratory study provides valuable insights into the genetic basis of facial morphology, it is important to emphasize that this study consists exclusively of orthodontic patients rather than a true population-based sample/cohort. Although strict exclusion criteria were applied—removing individuals with syndromic conditions, history of skull trauma, or cleft lip and palate—orthodontic seeking populations may still exhibit systematic differences in dentofacial and craniofacial characteristics compared to the general public. Consequently, while these findings establish an important baseline for the role of *FGFR2* polymorphisms in facial variation, caution should be exercised when generalizing these results to the broader population. Future large-scale, population-based studies are warranted to validate these genetic associations across more diverse and unselected cohorts.

## Conclusion

This exploratory study evaluating orthodontic patient’s children of European descent suggested that *FGFR2* polymorphisms may be involved with specific facial parameters, supporting a role of common genetic variants in *FGFR2* on facial development.

## Supplementary Information

Supplementary Material 1.

Supplementary Material 2.

Supplementary Material 3.

## Data Availability

The datasets used and/or analyzed during the current study are available fromthe corresponding author on reasonable request.
