# Supplementary material for: Exploring the association between genetic polymorphisms in FGFR2 and children’s facial morphology
Source: Head Face Med. 2026 Aug 15;22:67. doi: 10.1186/s13005-026-00649-3 (PMC13479409; doi:10.1186/s13005-026-00649-3)
Supplement: Supplementary file 1 [file 13005_2026_649_MOESM1_ESM.docx]

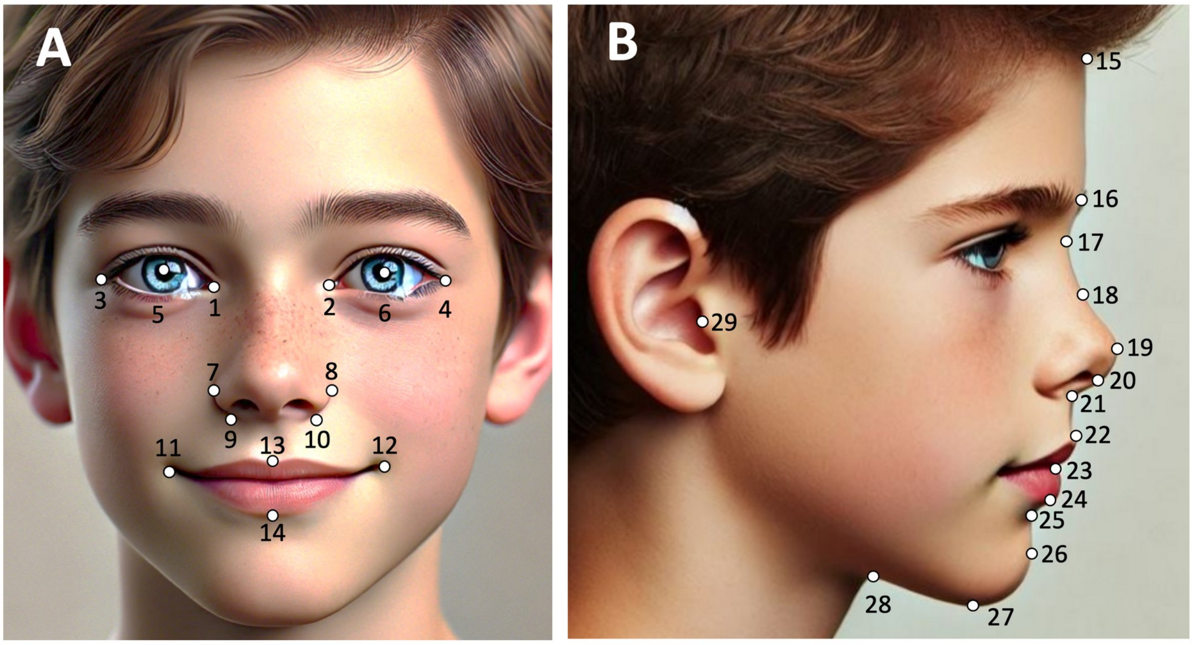
A
3
5
1
2
6
4
7
8
9
10
11
13
12
14
B
15
16
17
18
19
29
20
21
22
23
24
25
26
28
27
