# Supplementary material for: Exploring the association between genetic polymorphisms in FGFR2 and children’s facial morphology
Source: Head Face Med. 2026 Aug 15;22:67. doi: 10.1186/s13005-026-00649-3 (PMC13479409; doi:10.1186/s13005-026-00649-3)
Supplement: Supplementary file 2 [file 13005_2026_649_MOESM2_ESM.docx]

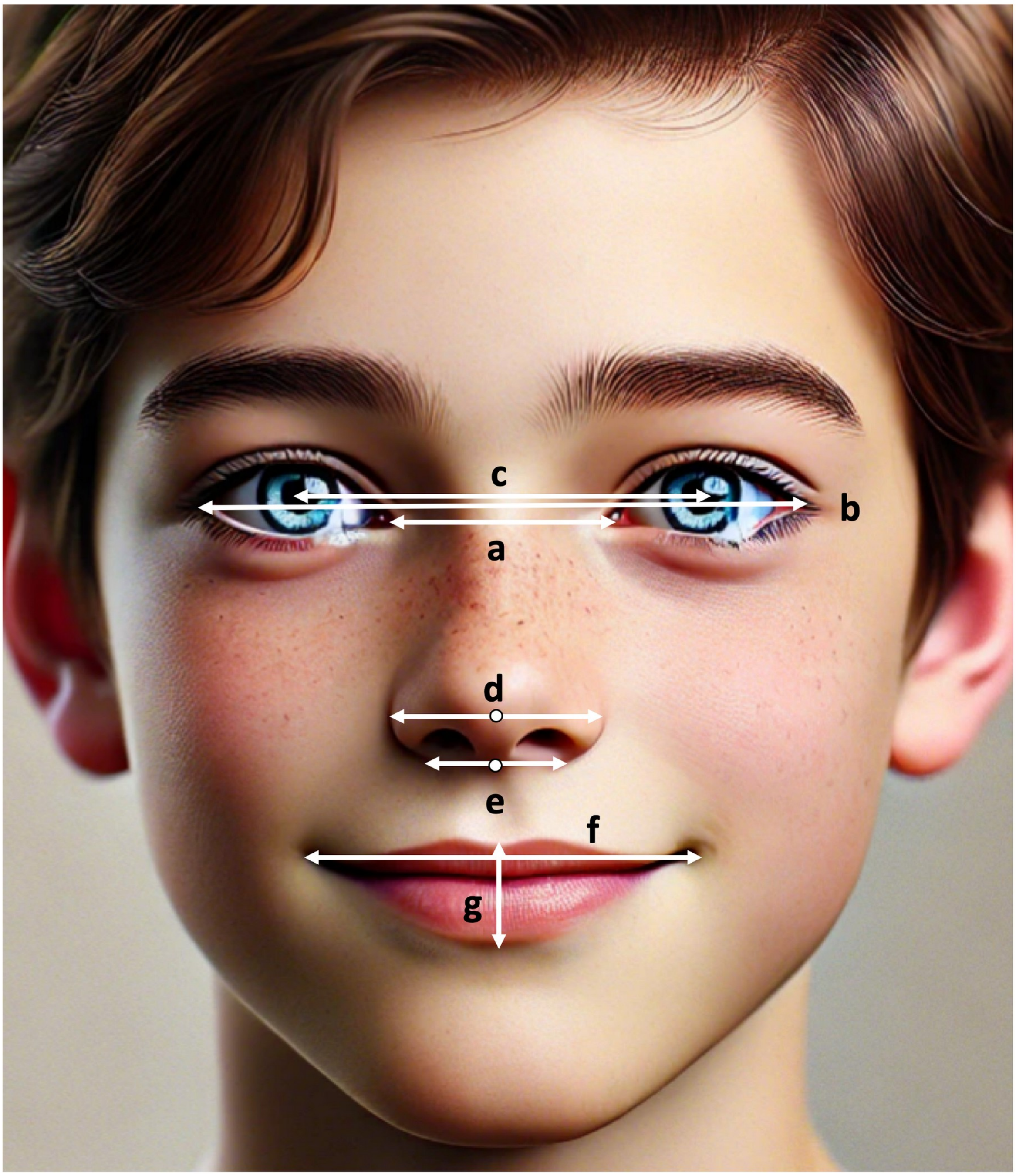
c
b
a
d
e
f
g
