# Supplementary material for: Exploring the association between genetic polymorphisms in FGFR2 and children’s facial morphology
Source: Head Face Med. 2026 Aug 15;22:67. doi: 10.1186/s13005-026-00649-3 (PMC13479409; doi:10.1186/s13005-026-00649-3)
Supplement: Supplementary file 3 [file 13005_2026_649_MOESM3_ESM.docx]

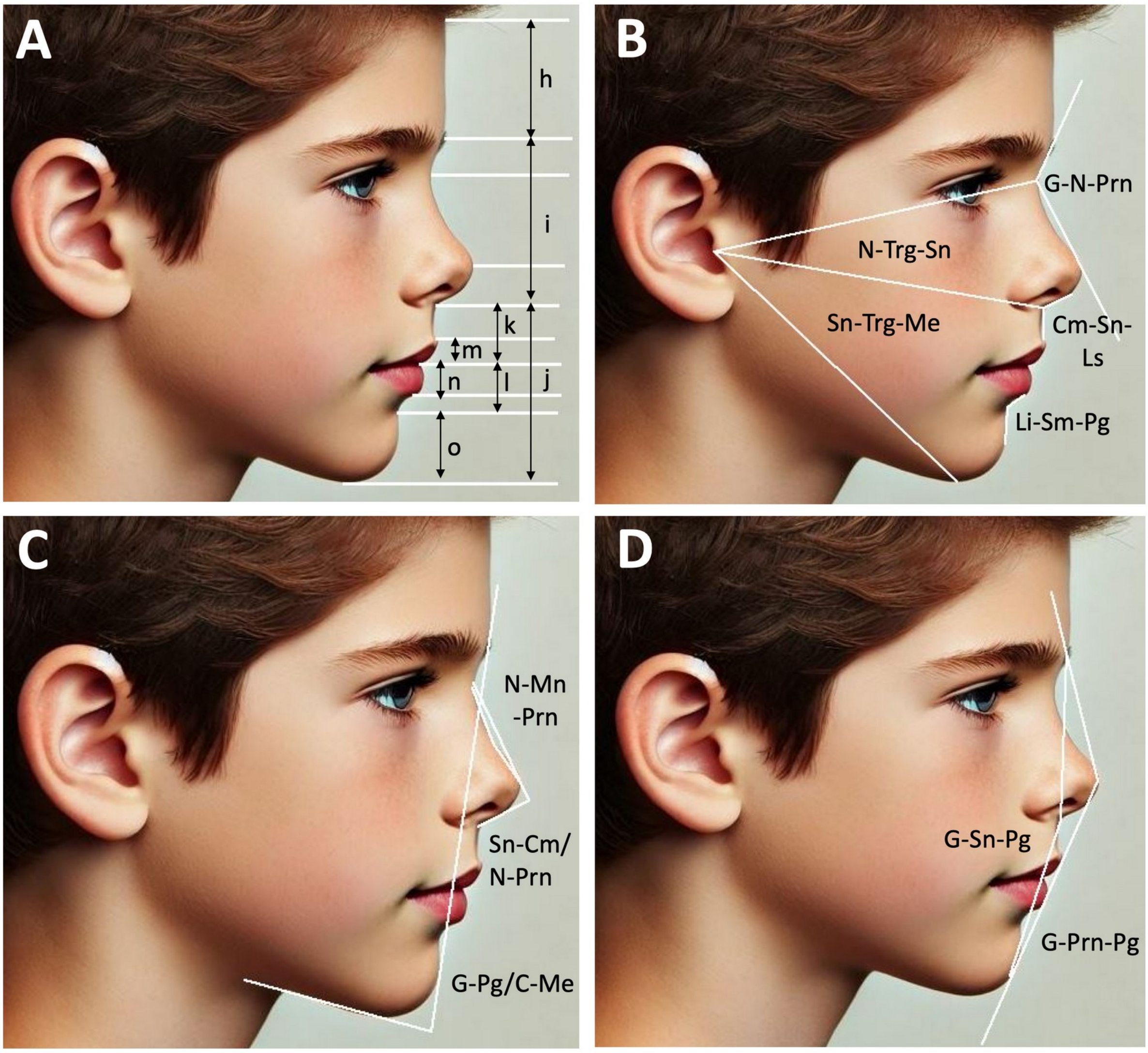
A
h
i
k
m
n
l
j
o
B
G-N-Prn
N-Trg-Sn
Sn-Trg-Me
Cm-Sn-
Ls
Li-Sm-Pg
C
N-Mn
-Prn
Sn-Cm/
N-Prn
G-Pg/C-Me
D
G-Sn-Pg
G-Prn-Pg
